# Association between supervisors’ behavior and wage workers’ job stress in Korea: analysis of the fourth Korean working conditions survey

**DOI:** 10.1186/s40557-017-0199-3

**Published:** 2017-10-11

**Authors:** Shin Uk Kang, Byeong Jin Ye, ByoungGwon Kim, Jung Il Kim, Jung Woo Kim

**Affiliations:** 1 Department of Occupational & Environmental Medicine, College of Medicine, Dong-A University, Busan, South Korea; 2 Gimhae Clinic Occupational Health Center, Inje University, Gimhae, South Korea

**Keywords:** Supervisor behavior, Job stress, Korean Working Conditions Survey, Feedback

## Abstract

**Background:**

In modern society, many workers are stressed. Supervisors’ support or behavior can affect the emotional or psychological part of the worker. The purpose of this study is to investigate the effect of supervisor’s behavior on worker’s stress.

**Methods:**

The study included 19,272 subjects following the assignment of weighted values to workers other than soldiers using data from the Fourth Korean Working Condition Survey. Supervisors’ behavior was measured using 5 items: “supervisor feedback regarding work,” “respectful attitude,” “good conflict-resolution ability,” “good work-related planning and organizational ability,” and the encouragement of participation in important decision making. Job stress was measured using 1 item: “I experience stress at work.” Multiple logistic regression analysis was performed to examine the effects of supervisors’ behavioral, general, occupational, and psychosocial characteristics on job stress in workers. Organizational characteristics associated with supervisors’ behavior were also analyzed.

**Results:**

The results showed that supervisors’ provision of feedback regarding work increased workers’ job stress (OR = 1.329, 95% CI = 1.203 ~ 1.468). When a supervisor respect workers (OR = 0.812, 95% CI = 0.722 ~ 0.913) or good at planning and organizing works (OR = 0.816, 95% CI: 0.732 ~ 0.910), workers’ job stress decreased. In particular, the two types of supervisor behaviors, other than feedback regarding work, were high in private-sector organizations employing less than 300 employees.

**Conclusion:**

Supervisors’ behavior influenced job stress levels in workers. Therefore, it is necessary to increase education regarding the effects of supervisors’ behavior on job stress, which should initially be provided in private-sector organizations with up to 300 employees.

## Background

Changes in supervisors’ behavior lead to changes in the workplace environment and could reduce aggression, poor manner to job, and physical and emotional strain in workers [1]. Moreover, supervisors’ leadership styles exert an impact on their performance, and leadership style, work environment, and job satisfaction are important factors affecting workers’ performance [2]. Furthermore, support from supervisors increases productivity indirectly by reducing the occurrence of presenteeism [3], and their communication styles and personality traits affect organizational productivity [4]. In addition, supervisors’ personalities and job stress levels have been associated with workers’ exposure to bullying in the workplace [5].

Supervisors’ behavior could influence workers’ job stress levels, and work-related stress is a serious problem that could exert an adverse effect on employees’ health [6, 7]. In particular, support from supervisors is an important factor affecting workers’ job satisfaction, and supervisors influence workers’ emotion [8]. In addition, supervisors’ behavior influences workers’ well-being, turnover intention, and job satisfaction [8–11]. For example, positive managerial behavior in the workplace could improve employees’ well-being [12], while destructive managerial leadership could exert an adverse effect [13]. Moreover, support from supervisors and coworkers, the maintenance of a relaxed atmosphere in the workplace, and increased respect for workers could reduce rates of presenteeism [14].

In previous studies, one of the many questions of the job stress questionnaire (e.g., Korean Occupational Stress Scale) used the question, "My boss helps me complete the task.", or there was a study on supervisor support [9, 15, 16]. For this reason, studies in the past have only been able to evaluate some of the various supervisors’ behaviors, and no studies have evaluated the supervisors’ behaviors using more detailed questionnaire.

The objective of the present study was to analyze the effect of supervisors’ behaviors using more detailed questionnaire, using data from the Fourth Korean Working Conditions Survey (KWCS), conducted in 2014.

## Methods

### Study subjects

**Fig. 1:**
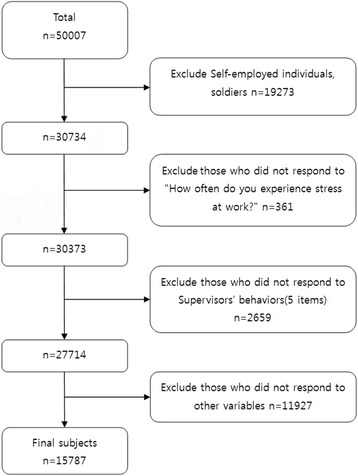
Subjects included in the current study

KWCS is a survey of Korean workers aged 15 and over to investigate the work environment and to identify exposure to risk factors according to job and industry type and risk factors according to employment type. Individual interviews were conducted. The 50,007 subjects included in the KWCS, data for 30,734 wage workers (excluding soldiers and self-employed individuals) and employers were analyzed in the present study. 27,714 subjects who answered the question "How often do you experience stress at work?" And the questions about supervisors’ behaviors were selected. For the other variables, 15,787 subjects were finally selected, excluding those who did not respond or rejected (Fig. 1). After weighting, the weighted frequency was 19,064 and the data were analyzed.

### Variables

#### General characteristics

Subjects’ general characteristics, included age, sex, education level, average monthly income, General health condition. The unit of monthly income is 10,000 Korean won. Educational levels were categorized as low for subjects educated to high school level or lower and high for subjects educated to college level or higher. Job stress was set to ‘Low’ for 1 ~ 2 points and ‘High’ for 3 ~ 5 points. General health condition is very good, good, normal is defined as Good, bad, and very bad as Bad.

#### Occupational characteristics

Occupational characteristics included the current number of employees, sector (public or private), occupation type, employment status, shift work, and working hours. Organizations were classified into 2 groups: those with up to 300 employees and those with more than 300 employees. Privately owned companies were classified as private-sector organizations, while central and local government offices; public institutions; and state-owned schools, hospitals, and universities were classified as public-sector organizations. Occupation types were categorized as white collar (e.g., supervisors, professionals, mechanics, semiprofessionals, and office workers) or service/sales workers (e.g., service workers; sales workers) or blue collar (e.g., skilled workers in agriculture, forestry and fishing; technicians; related technical workers; device or machine operation and assembly workers, and simple laborers) occupations. Employment status was classified as permanent or temporary, and shift pattern (shift work or non-shift work) was included as a variable.

#### Psychosocial risk factors

Psychosocial risk factors for job stress were categorized into the following 5 categories: high job demand, insufficient job control, inadequate social support, job insecurity, and lack of reward. The questionnaires used in this study are listed in Appendix. Each item was scored, and scores were classified as high or low based on the median.

#### Supervisors’ behavior

Supervisors’ behavior was measured using the following 5 items: my supervisor provides feedback regarding my work (Feedback), my supervisor respects me personally (Respectful behavior), my supervisor is good at resolving conflict (Good conflict abilities), my supervisor is good at planning and organizing work (Good at planning), and my supervisor encourages me to contribute to important decisions (Participation in decisions). The response options were “yes” or “no” for all items.

#### Job stress

The frequency with which workers experienced job stress was measured using the following item: How often do you experience stress at work? Response options were as follows: 5 = always, 4 = most of the time, 3 = sometimes, 2 = seldom, 1 = never.

#### Statistical analysis

Chi-square was performed to determine differences in subjects’ general and occupational characteristics according to supervisors’ behavior. Multiple logistic regression analysis was performed to examine subjects’ general characteristics and occupational characteristics and identify the effects of supervisors’ behavior on workers’ job stress levels. All analyses were performed using SPSS ver. 23.0 (Chicago, IL, USA).

## Results

### General characteristics according to occupational characteristics

The study included 19,064 subjects (9770 men, 9294 women). There were more people in organizations with less than 300 employees, and more people worked in the private sector than in the public sector. Between age and number of employees, age and public/private sector showed significant differences (*p* < 0.001). In organizations with less than 300 employees, the number of people aged between 41 and 50 was high, while those with more than 300 workers were between 31 and 40. There were many workers in the 41–50 age group in both private and public sectors. The proportion of male and high education level workers was significantly higher in organizations with more than 300 employees than that of organizations with up to 300 employees (*p* < 0.001). In the private sector, the proportion of men and high education level workers was high (*p* < 0.001). There was no difference in the general health condition according to the number of workers (*p* = 0.060), and more people in the public sector answered that they had good general health condition (*p* < 0.001). Between monthly income and number of employees, monthly income and public/private sector were significantly different (*p* < 0.001). In organizations with more than 300 employees, the proportion of people with income over 3 million won was high. The proportion of high job stress of the private sector was significantly higher than that of high job stress of the public sector (*p* < 0.001) (Table 1).

**Table 1:** Subjects’ General characteristics according to Occupational characteristics

		Number of employees			Sector		
	Total	<300 (%)	≥300 (%)		Public (%)	Private (%)	
Total	19064^a^ (100%)	17436 (91.5)	1628 (8.5)		2399 (12.6)	16665 (87.4)	
Age							
≤ 30	3302 (17.3)	3040 (17.4)	262 (16.1)	<0.001^b^	221 (9.2)	3081 (18.5)	<0.001^b^
31 ~ 40	5201 (27.3)	4626 (26.5)	576 (35.4)		580 (24.2)	4621 (27.7)	
41 ~ 50	5480 (28.7)	5025 (28.8)	455 (27.9)		750 (31.3)	4731 (28.4)	
51 ~ 60	3383 (17.7)	3086 (17.7)	297 (18.2)		448 (18.7)	2935 (17.6)	
≥ 61	1698 (8.9)	1660 (9.5)	38 (2.3)		400 (16.7)	1297 (7.8)	
Sex							
Male	9770 (51.2)	8582 (49.2)	1188 (73.0)	<0.001^b^	1149 (47.9)	8622 (51.7)	<0.001^b^
Female	9294 (48.8)	8854 (50.8)	440 (27.0)		1250 (52.1)	8044 (48.3)	
Educational level							
High school or lower	8928 (46.8)	8406 (48.2)	523 (32.1)	<0.001^b^	800 (33.3)	8129 (48.8)	<0.001^b^
College or higher	10136 (53.2)	9031 (51.8)	1105 (67.9)		1599 (66.7)	8536 (51.2)	
General health condition							
Good	18525 (97.2)	16931 (97.1)	1594 (97.9)	0.060^b^	2290 (95.5)	16235 (97.4)	<0.001^b^
Bad	539 (2.8)	505 (2.9)	34 (2.1)		109 (4.5)	430 (2.6)	
Income							
< 100	1906 (10.0)	1881 (10.8)	25 (1.5)	<0.001^b^	504 (21.0)	1402 (8.4)	<0.001^b^
100 ~ 199	6475 (34.0)	6282 (36.0)	192 (11.8)		408 (17.0)	6066 (36.4)	
200 ~ 299	5737 (30.1)	5289 (30.3)	448 (27.5)		618 (25.8)	5119 (30.7)	
≥ 300	4946 (25.9)	3984 (22.8)	962 (59.1)		868 (36.2)	4078 (24.5)	
Job stress							
High	14443 (75.8)	13174 (75.6)	1269 (77.9)	0.031^b^	1711 (71.3)	12733 (76.4)	<0.001^b^
Low	4621 (24.2)	4262 (24.4)	359 (22.1)		688 (28.7)	3933 (23.6)	

^a^weighted frequency

^b^Based on the chi-square test

### Supervisors’ behavior according to occupational characteristics

The proportion of people who answered “yes” to the “my supervisor provides feedback regarding my work,” “my supervisor respects me personally,” “my supervisor is good at resolving conflict,” “my supervisor is good at planning and organizing work,” and “my supervisor encourages me to contribute to important decisions” items, which reflected the quality of supervisors’ behavior, in organizations with more than 300 employees were significantly higher relative to those observed for organizations with up to 300 employees (*p* < 0.001).

The proportion of public-sector organizations who responded in the affirmative for all items pertaining to supervisors’ behavior was significantly greater relative to that observed for private-sector organizations (*p* < 0.001). However, answer to “my supervisor provides feedback on my work” did not differ significantly between public- and private-sector organizations (*p* = 0.496) (Table 2).

**Table 2:** Supervisors’ behaviors according to Occupational characteristics

		Number of employees		Sector	
	Total	<300 (%)	≥300 (%)	Public (%)	Private (%)
Total	19064^a^ (100%)	17436 (91.5)	1628 (8.5)	2399 (12.6)	16665 (87.4)
Feedback					
Yes	14380 (75.4)	12991 (74.5)	1388 (85.3)^***^	1823 (76.0)	12557 (75.3)
No	4684 (24.6)	4445 (25.5)	240 (14.7)	576 (24.0)	4108 (24.7)
Respectful behavior					
Yes	15880 (83.3)	14382 (82.5)	1499 (92.1)^***^	2120 (88.4)	13761 (82.6)^***^
No	3184 (16.7)	3055 (17.5)	129 (7.9)	279 (11.6)	2904 (17.4)
Good conflict abilities					
Yes	14067 (73.8)	12661 (72.6)	1406 (86.4)^***^	1902 (79.3)	12165 (73.0)^***^
No	4997 (26.2)	4775 (27.4)	221 (13.6)	497 (20.7)	4500 (27.0)
Good at planning					
Yes	14536 (76.2)	13111 (75.2)	1425 (87.5)^***^	1955 (81.5)	12581 (75.5)^***^
No	4528 (23.8)	4325 (24.8)	203 (12.5)	444 (18.5)	4084 (24.5)
Participation in decisions					
Yes	12050 (63.2)	10766 (61.7)	1285 (78.9)^***^	1646 (68.6)	10404 (62.4)^***^
No	7014 (36.8)	6671 (38.3)	343 (21.1)	753 (31.4)	6261 (37.6)

^***^ *p* < 0.001

^a^weighted frequency

### Psychosocial risk factors of study subjects by Occupational characteristics

Organizations with more than 300 employees had high job demands than organizations with less than 300 employees, the private sector has a high proportion of high job demand than the public sector (*p* < 0.001). Organizations with more than 300 employees and the public sector had high job control (*p* < 0.001). The high social support proportion was higher in the organizations with more than 300 employees, and the high social support proportion was high in the public sector, showing a significant difference (*p* < 0.001). Job insecurity and the number of employees showed no significant difference (*p* = 0.172) (Table 3).

**Table 3:** Psychosocial risk factors of study subjects by Occupational characteristics

		Number of employees		Sector	
	Total	<300 (%)	≥300 (%)	Public (%)	Private (%)
Total	19064^a^ (100%)	17436 (91.5)	1628 (8.5)	2399 (12.6)	16665 (87.4)
Job demand					
High	8238 (43.2)	7398 (42.4)	840 (51.6)^***^	768 (32.0)	7470 (44.8) ^***^
Low	10826 (56.8)	10038 (57.6)	788 (48.4)	1630 (68.0)	9196 (55.2)
Job control					
High	10451 (54.8)	9476 (54.3)	975 (59.9) ^***^	1393 (58.1)	9059 (54.4) ^***^
Low	8613 (45.2)	7960 (45.7)	653 (40.1)	1006 (41.9)	7607 (45.6)
Social support					
High	10784 (56.6)	9557 (54.8)	1227 (75.4) ^***^	1527 (63.7)	9257 (55.5) ^***^
Low	8280 (43.4)	7879 (45.2)	401 (24.6)	872 (36.3)	7408 (44.5)
Job insecurity					
High	6072 (31.8)	5578 (32.0)	494 (30.3)	627 (26.1)	5445 (32.7)^***^
Low	12992 (68.2)	11858 (68.0)	1134 (69.7)	1772 (73.9)	11221 (67.3)
Lack of reward					
High	6972 (36.6)	6122 (35.1)	850 (52.2)^***^	1216 (50.7)	5757 (34.5)^***^
Low	12092 (63.4)	11314 (64.9)	778 (47.8)	1183 (49.3)	10908 (65.5)

^***^ *p* < 0.001

^a^weighted frequency

### Multiple logistic regression analysis of factors affecting job stress

The results showed that workers who reported that supervisors provided work-related feedback exhibited high job stress levels (OR = 1.329, 95% CI = 1.203 ~ 1.468). However, those who reported that a supervisor does respectful behaviors (OR = 0.812, 95% CI = 0.722 ~ 0.913) and planning and organizing work exhibited low job stress levels (OR = 0.816, 95% CI = 0.732 ~ 0.910). In addition, job stress did not differ significantly according to conflict solving abilities or participation in decision-making (Table 4).

**Table 4:** Multiple logistic regression analysis of factors affecting job stress

Variables	Unadjusted		Adjusted	
	OR^b^	95% CI^c^	OR^b^	95% CI^c^
Feedback				
No	1.00		1.00	
Yes	1.404	1.297 ~ 1.520	1.329	1.203 ~ 1.468
Respectful behavior				
No	1.00		1.00	
Yes	1.074	0.984 ~ 1.173	0.812	0.722 ~ 0.913
Good conflict abilities				
No	1.00		1.00	
Yes	1.101	1.022 ~ 1.186	0.977	0.881 ~ 1.083
Good at planning				
No	1.00		1.00	
Yes	1.075	0.996 ~ 1.161	0.816	0.732 ~ 0.910
Participation in decisions				
No	1.00		1.00	
Yes	1.308	1.221 ~ 1.401	1.109	0.999 ~ 1.230

adjusted by job demand, job control, social support, job insecurity, lack of reward, sex, shift work, sector, income, age, education level, type of employment, number of employees, working hours, occupation, general health condition

^b^odds ratio, ^c^confidence interval

## Discussion

This study aimed to examine the effects of supervisors’ behavior on job stress in Korean workers. In this study, Feedback, Respectful behaviors, and Good at planning were significant results with job stress. When a supervisor gives feedback, work stress of worker was high. When supervisors respect workers or organize their work well Workers’ stress was low.

In this study, Feedback, Respectful behaviors and Good at planning showed significant results.

First, the results of the current study showed that supervisors’ provision of work-related feedback increased workers’ stress levels. This finding was consistent with those of a previous study indicating that supervisors’ provision of work-related feedback increased job stress in workers [6]. However, another study showed that supervisors’ provision of feedback in an appropriate environment reduced work-related stress in workers [17]. Numerous studies have shown that the environment in which supervisors provided feedback exerted both positive and negative effects on employees’ job stress levels [17–20]. Moreover, supervisors’ feedback was shown to influence both positive factors, such as person-organization fit and organizational commitment, and negative factors, such as role stress or burnout [20]; in addition, feedback environments exerted either positive or negative effects according to the characteristics of the organization or individual workers [18]. In previous studies, defensive or self-deceitful workers were more affected by bad feedback than good feedback [21]. According to Steelman, “favorable feedback” is expressed as the frequency of receiving positive feedback such as compliments from the perspective of the employee receiving the feedback, and “unfavorable feedback” is the frequency of complaints from managers or colleagues And the frequency of negative feedback such as criticism [22]. Both “favorable feedback” and “unfavorable feedback” are important in reducing the deviance of the worker in terms of the feedback environment [17].

Second, when a supervisor respect workers, the worker’s stress appeared to be low. In previous studies, there have been few studies on the relationship between supervisors’ respectful behaviors and worker’s job stress. According to Cobb, social support consists of three components: information that they are being cared for and loved, information that they are being esteemed, and valued, and information that they belong to one of the group members. In this study, the meaning of ‘Respectful behavior’ means ‘my supervisor respects me personally’ and is one of the components of social support according to the definition of Cobb [23]. There are studies that suggest that social support affects worker’s job stress [23, 24].

Third, when the supervisor’s planning ability was good, the stress level was low. The meaning of “planning” in this study means to organize and plan work well. In previous studies, there have been few study on the relationship between supervisors’ planning ability and job stress. However, there have been studies that have increased job stress when work overload has increased [25, 26]. Supervisors are in an important position to manage role stress. They can influence role overload by determining the scope of work of an employee, and can monitor the worker’s impact on a role. When workers experience quantitative overload, they can help workers by analyzing their work, providing training that helps them work, or introducing workers who do a similar job. Or to help workers who are experiencing quantitative overload by distributing part of the work to another worker or by putting in another worker [27]. With these organizing or planning approaches, managers can reduce psychological strain by preventing worker overload [28].

The present study was subject to some limitations. For example, although the KWCS, which was a cross-sectional study, demonstrated an association between supervisors’ behavior and workers’ stress, the inference of causality in this relationship was limited. In addition, psychosocial stress, outcome variables, and independent variables were measured via a self-report questionnaire in the current study, which could have led to response biases. Moreover, as work-related stress was not measured using a standardized instrument, such as the Korean Occupational Stress Scale, in the present study, the precision of the measurement of this variable was limited. Despite these limitations, the present study clarified the association between supervisors’ behavior and job stress in workers, using nationally representative data collected via the 2014 KWCS.

## Conclusion

According to the present study, supervisors’ behaviors affected on job stress of Korean workers. Therefore, supervisors working in private-sector organizations with fewer than 300 employees should consider the characteristics of workers and be provided appropriate feedback education, receive training in respectful behaviors and organizational skills, which could contribute to the reduction of job stress in workers.

## Abbreviations

CI: Confidence interval
KWCS: Korean Working Condition Survey
